# Intake of an Obesogenic Cafeteria Diet Affects Body Weight, Feeding Behavior, and Glucose and Lipid Metabolism in a Photoperiod-Dependent Manner in F344 Rats

**DOI:** 10.3389/fphys.2018.01639

**Published:** 2018-11-26

**Authors:** Roger Mariné-Casadó, Cristina Domenech-Coca, Josep Maria del Bas, Cinta Bladé, Lluís Arola, Antoni Caimari

**Affiliations:** ^1^Eurecat, Centre Tecnològic de Catalunya, Technological Unit of Nutrition and Health, Reus, Spain; ^2^Nutrigenomics Research Group, Department of Biochemistry and Biotechnology, Universitat Rovira i Virgili, Tarragona, Spain

**Keywords:** photoperiod, circannual rhythms, cafeteria diet, metabolic syndrome, feeding behavior, glucose homeostasis

## Abstract

We previously demonstrated that chronic exposure to different photoperiods induced marked variations in several glucose and lipid metabolism-related parameters in normoweight Fischer 344 (F344) rats. Here, we examined the effects of the combination of an obesogenic cafeteria diet (CAF) and the chronic exposure to three different day lengths (L12, 12 h light/day; L18, 18 h light/day; and L6, 6 h light/day) in this rat strain. Although no changes were observed during the first 4 weeks of adaptation to the different photoperiods in which animals were fed a standard diet, the addition of the CAF for the subsequent 7 weeks triggered profound physiologic and metabolic alterations in a photoperiod-dependent manner. Compared with L12 rats, both L6 and L18 animals displayed lower body weight gain and cumulative food intake in addition to decreased energy expenditure and locomotor activity. These changes were accompanied by differences in food preferences and by a sharp upregulation of the orexigenic genes *Npy* and *Ghsr* in the hypothalamus, which could be understood as a homeostatic mechanism for increasing food consumption to restore body weight control. L18 rats also exhibited higher glycemia than the L6 group, which could be partly attributed to the decreased pAkt2 levels in the soleus muscle and the downregulation of *Irs1* mRNA levels in the gastrocnemius muscle. Furthermore, L6 animals displayed lower whole-body lipid utilization than the L18 group, which could be related to the lower lipid intake and to the decreased mRNA levels of the fatty acid transporter gene *Fatp1* observed in the soleus muscle. The profound differences observed between L6 and L18 rats could be related with hepatic and muscular changes in the expression of circadian rhythm-related genes *Cry1*, *Bmal1*, *Per2*, and *Nr1d1.* Although further research is needed to elucidate the pathophysiologic relevance of these findings, our study could contribute to emphasize the impact of the consumption of highly palatable and energy dense foods regularly consumed by humans on the physiological and metabolic adaptations that occur in response to seasonal variations of day length, especially in diseases associated with changes in food intake and preference such as obesity and seasonal affective disorder.

## Introduction

Seasonal variations in environmental factors provide crucial information to animals, allowing them to adapt their organism through changes in many physiological and behavioral parameters (Paul et al., 2008; Helm et al., 2013). Despite human isolation from environmental annual changes as a consequence of the appearance of heat and air-conditioning systems and artificial light in developed economies (Kanikowska et al., 2014), several studies have proven that human patterns of birth, death, or disease are season-dependent (Foster and Roenneberg, 2008). In addition, body fat mass accretion and circulating cholesterol, triglycerides, leptin, glucose, and insulin levels can be significantly increased in winter, accounting for higher rates of cardiac events in this season (Pell and Cobbe, 1999; Reilly and Peiser, 2006; Haus, 2007; Liang, 2007; Kanikowska et al., 2014). For example, in a study performed on 1,202 Japanese male workers, Kamezaki et al. (2010) reported higher systolic and diastolic blood pressure and increased fasting blood glucose levels in winter than in summer, concluding marked seasonal variation in the prevalence of metabolic syndrome (MetS), which is defined as a cluster of interconnected risk factors—obesity, insulin resistance, dyslipidemia, and hypertension—that increase the risk of cardiovascular disease (CVD) (Cornier et al., 2008; Kaur, 2014). These authors also described that the higher MetS incidence observed during winter was associated with a moderate increase in insulin resistance (Kamezaki et al., 2013).

Among the different environmental conditions that vary throughout the year, some, such as temperature and food availability, are considered low predictive factors since they do not display specific timing or magnitude (Weil et al., 2015). However, seasonal variations in day length are the main environmental cue that offers a highly predictive signal of the correct time of year (Weil et al., 2015). Due to the possibility of constantly controlling the photoperiod, laboratory animals have emerged as a valuable model to gain knowledge on how humans respond to seasonal variations in day length. The Fischer 344 (F344) rat strain is a clear example of an animal model that has become relevant in the study of circannual rhythms (Heideman and Sylvester, 1997; Heideman et al., 2000; Shoemaker and Heideman, 2002; Ross et al., 2011; Tavolaro et al., 2015). Our group recently described that normoweight F344 rats exposed to different photoperiods for 14 weeks displayed profound metabolic changes, highlighting the importance that the seasonal changes in day length can have on health and suggesting these rats as a promising animal model with which to study glucose- and lipid-related pathologies that are influenced by seasonal variations, such as obesity, MetS, and CVD (Mariné-Casadó et al., 2018). These effects were more evident in rats held under a short day (SD) photoperiod (6 h of light), which showed an insulin resistance-like phenotype, as evidenced by increased circulating glucose levels, a vast downregulation of the muscular downstream postreceptor target of insulin Akt serine/threonine kinase 2 (Akt2), and decreased gene expression of the hepatic glucose transporter *Glut2* and the muscular insulin receptor substrate 1 (*Irs1*) (Mariné-Casadó et al., 2018). Our results partly agree with those reported by Tashiro et al. (2017), which revealed that C57BL/6J mice exposed to SD conditions (8 h of light) for 3 weeks displayed higher circulating glucose levels, which were explained by the reduced glucose transporter 4 (GLUT4) protein levels in the gastrocnemius muscle. These authors also demonstrated that this animal model exhibited increased body weight, fat mass accretion, and sucrose intake, and a depression-like behavior, partly mirroring the seasonal affective disorder (SAD) that occurs in humans mainly in winter (Otsuka et al., 2014, 2015).

There is little information regarding the effects of chronic exposure to different photoperiods under obesogenic conditions. In male obese Zucker rats, which display genetic obesity and type 2 diabetes due to deficiencies in leptin receptor, Larkin et al. (1991) described that exposure to a long day (LD) photoperiod (14 h of light) for 9 weeks increased the insulin circulating levels, lean body mass, and energy efficiency compared with exposure to an SD photoperiod (10 h of light). In addition, obese Zucker rats exhibited a more pronounced response to photoperiod exposure than their lean counterparts. Nevertheless, highly caloric palatable diet-induced obese models are more representative of the etiology of obesity and MetS in modern societies (Li et al., 2008), since genetics contributes to a lesser extent to the development of obesity and its comorbidities than sedentary lifestyles combined with excess energy intake (Hruby and Hu, 2015). In this sense, Togo et al. (2012) reported that F344 rats held under LD conditions (16 h of light) and fed a high-fat diet (HFD) for 3 weeks displayed increased body weight, epididymal adipose tissue, and leptin levels compared with animals exposed to an SD (8 h of light). In another experiment performed under the same photoperiodic conditions, it was demonstrated that photoperiod regulated feeding behavior, which was evidenced by a higher preference for a low-fat, high-carbohydrate (CH) diet than for the HFD in LD F344 rats, an effect that was not observed in SD animals (Togo et al., 2012). In contrast, Ross et al. (2015) described that the photoperiodic regulation of different parameters, such as the stimulation of fat mass in LD photoperiods, was dampened after HFD feeding, whereas lean mass and other photoperiod-responsive parameters were unaffected by HFD exposure.

Among the high caloric diets, the CAF, which contains a variety of highly palatable and energy dense foods prevalent in Western society, has become a more useful choice than a HFD to resemble metabolic and eating behavioral processes underlying diet-induced human obesity and MetS (Sampey et al., 2011; Lalanza et al., 2014; Cigarroa et al., 2016). Nevertheless, the effects of the combination of a CAF with different photoperiod exposures on physiology and metabolic homeostasis in F344 rats have not yet been examined. In the present study, we hypothesized that physiologic- and metabolic-related parameters of CAF-fed obese F344 rats would be influenced by chronic exposure to different day lengths. Therefore, the aim of the present work was to study the photoperiodic changes in a variety of physiological and metabolic outputs of F344 rats fed a CAF.

## Materials and Methods

### Animals

Thirty 8-week-old male F344 rats (Charles River Laboratories, Barcelona, Spain) were housed in pairs in cages at 22°C and submitted to three different light schedules for 11 weeks to mimic seasonal day lengths: SD photoperiod [*n* = 10, L6, 6 h light – from Zeitgeber times (ZTs) 0 to 6 – and 18 h darkness – from ZTs 6 to 24], normal day (ND) photoperiod [*n* = 10, L12, 12 h light – from ZTs 0 to 12 – and 12 h darkness – from ZTs 12 to 24], and LD photoperiod (*n* = 10, L18, 18 h light – from ZTs 0 to 18 – and 6 h darkness – from ZTs 18 to 24). The three groups were subjected to a 4-week adaptation period in which animals were fed a standard diet (STD) *ad libitum* (2.90 kcal g^-1^; Teklad Global 14% Protein Rodent Diet 2014, ENVIGO, Sant Feliu de Codines, Barcelona, Spain). After this period, rats were switched to a CAF for 7 weeks. The CAF contained bacon, biscuit with pâté, and biscuit with cheese, carrots, muffins, and milk with sugar (22 g/L). Its caloric distribution was 58.1% CH, 31.9% lipid, and 10.0% protein, as previously described (Caimari et al., 2017). During the entire study, rats had free access to chow and water, and body weight and food intake data were recorded once a week. After 11 weeks, animals were sacrificed by decapitation at ZT1, being deprived of food for 1 h. Blood was collected, and serum was obtained by centrifugation and stored at -80°C until analysis. The liver, hypothalamus, and soleus and gastrocnemius muscles were rapidly weighed, frozen in liquid nitrogen, and stored at -80°C for further analysis. The Animal Ethics Committee of the Universitat Rovira i Virgili (Tarragona, Spain) approved all procedures.

### Body Composition Analysis

Lean and fat measurements (in grams and percentage of body weight) were performed 1 week before sacrifice using an EchoMRI-700^TM^ device (Echo Medical Systems, L.L.C., Houston, TX, United States).

### Indirect Calorimetry

Indirect calorimetry analyses were performed 2 weeks before sacrifice using the OxyletPro^TM^ System (PANLAB, Cornellà, Spain). After receiving treatment at ZT0, rats were transferred to an acrylic box (Oxylet LE 1305 Physiocage, PANLAB) with free access to water and food. After an acclimation period of 3 h, oxygen consumption (VO_2_) and carbon dioxide production (VCO_2_) were measured every 9 min by an O_2_ and CO_2_ analyzer (Oxylet LE 405 gas analyzer, PANLAB) at a constant flow rate of 600 mL/min (Oxylet LE 400 air supplier, PANLAB). At each measure, the program Metabolism 2.1.02 (PANLAB, Cornellà, Spain) calculated the respiratory quotient (RQ) as the VCO_2_/VO_2_ ratio and energy expenditure (EE) as VO_2_ × 1.44 × [3.815 + (1.232 × RQ)] (kcal/day/kg^0.75^) according to the Weir formula (Weir, 1949). Fat and CH oxidation rates were calculated using the VCO_2_ and the VO_2_ measures applying the Frayn stoichiometric equations (Frayn, 1983), which define fat oxidation rates as 1.67 × VO_2_ – 1.67 × VCO_2_ – 1.92 *n* (g min^-1^) and CH oxidation rates as 4.55 × VCO_2_ – 3.21 × VO_2_ – 2.87 *n* (g min^-1^). A nitrogen excretion rate (*n*) of 135 μg kg^-1^ min^-1^ was assumed (Carraro et al., 1990). Finally, the fat and CH oxidation energy (in kJ min^-1^) was obtained by using the Atwater general conversion factor. The fat and CH rates were multiplied by 37 and 16, respectively (Bircher and Knechtle, 2004).

### Serum Analysis

Glucose, total cholesterol, and triglycerides (QCA, Barcelona, Spain), phospholipids (Spinreact, Girona, Spain), and non-esterified free fatty acids (NEFAs) (WAKO, Neuss, Germany) were analyzed by enzymatic colorimetric assays. Serum insulin and glucagon levels were analyzed using a rat insulin ELISA kit (Millipore, Barcelona, Spain) and a rat glucagon ELISA kit (Cusabio Biotech, Wuhan, China), respectively.

### Serum Extraction and ^1^H Nuclear Magnetic Resonance (NMR) Analysis of Metabolite Analysis

Serum metabolites were extracted as previously described (Mariné-Casadó et al., 2018). ^1^H-NMR analysis was performed following the procedure described by Vinaixa et al. (2010).

### Gene Expression Analysis

Total RNA extraction, cDNA synthesis, and real-time quantitative PCR in the hypothalamus, liver, and gastrocnemius, and soleus muscles were performed as previously detailed (Mariné-Casadó et al., 2018). The primers used for the different genes are described in Supplementary Table 1 and were obtained from Biomers.net (Ulm, Germany). The relative expression of each mRNA level was calculated as a percentage of the L12 group using the -2^ΔΔCt^ method (Livak and Schmittgen, 2001) with β-*actin*, *Ppia*, *Hprt*, and *Tfrc* genes as references.

### Western Blot Analysis

Total and phosphorylated (p) AMP-activated protein kinase [AMPK and (p)-AMPK] and Akt2 and (p)-Akt2 protein levels in the liver and soleus and gastrocnemius muscles were measured by Western blot analysis as previously described (Mariné-Casadó et al., 2018).

### Statistical Analysis

Data are expressed as the mean ± standard error of the mean (SEM) (*n* = 8–10). The effect of photoperiod on the evolution of body weight gain; cumulative caloric intake; and the cumulative intake of CH, lipids, and proteins was analyzed by repeated measures (RMs) analysis of variance (ANOVA) with time as a within-subject factor and photoperiod as a between-subject factor. When the interaction between time and photoperiod (*Pxt*) was statistically significant, one-way ANOVA followed by Duncan’s *post hoc* test was used to determine significant differences among the three groups in each point. One-way ANOVA and Duncan’s *post hoc* test were also used to determine the photoperiod effects on biometric, serum, and metabolic parameters; fiber intake; and specific food items of the CAF. Student’s *t*-test was also used for single statistical comparisons. Grubbs’ test was used to detect outliers, which were discarded before subsequent analyses. All statistical tests were performed with the statistical software IBM SPSS Statistics 25.0 (SPSS, IBM Corp. Armonk, NY, United States). The level of statistical significance was set at bilateral 5%.

Principal component analysis (PCA) and partial least squares discriminant analysis (PLS-DA) were performed after data normalization and autoscaling using MetaboAnalyst 3.0 software (Xia et al., 2015).

## Results

### Exposure to Both Short and Long Photoperiods Combined With CAF Feeding Altered Food Intake-Related Parameters and Body Weight

**FIGURE 1:**
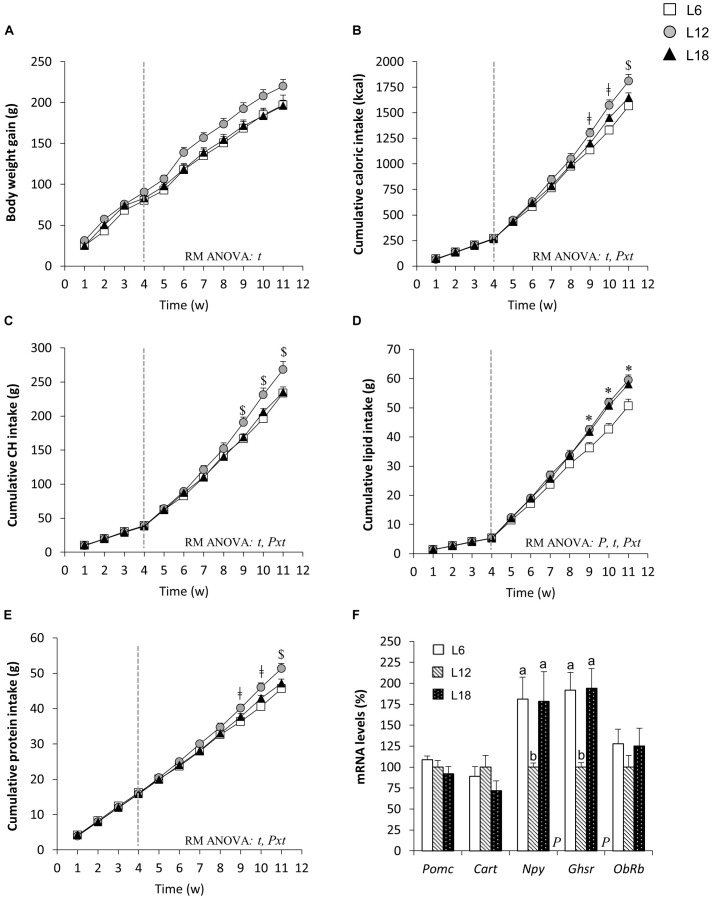
Body weight gain **(A)**, cumulative caloric intake **(B)**, CH intake **(C)**, lipid intake **(D)**, protein intake **(E)**, and hypothalamic mRNA levels of genes related to food intake control **(F)** in male F344 rats exposed to three different photoperiods for 11 weeks and fed a cafeteria diet for the last 7 weeks. The end of the 4-week adaptation period is represented by a vertical dotted line. Data are expressed as the mean ± SEM (*n* = 8–10). *P*, photoperiod effect; *t*, time effect; *Pxt*, photoperiod × time interaction effect (*p* < 0.05, RM ANOVA). $*p* < 0.05 L12 versus L18 and L6 groups; ^Δ^*p* < 0.05 L12 versus L18 group; ǂ*p* < 0.05 L12 versus L6 group; ^∗^*p* < 0.05 L6 versus L12 and L18 groups. ^ab^Mean values with unlike letters were significantly different among groups (one-way ANOVA and Duncan’s *post hoc* test). CH, carbohydrates; *Cart*, cocaine and amphetamine-regulated transcript; *Ghsr*, ghrelin receptor; *Npy*, neuropeptide Y; *ObRb*, long-form leptin receptor; *Pomc*, proopiomelanocortin.

Exposure to different photoperiods during the 4-week adaptation period did not produce significant changes in body weight gain, cumulative food intake, or macronutrient consumption among groups (Figures 1A–E). However, the shift to the CAF combined with exposure to different day lengths for seven additional weeks triggered a photoperiod-dependent response in all parameters related with food intake (*p* < 0.05, photoperiod × time interaction, RM ANOVA) (Figures 1B–E). Thus, L6 rats displayed significantly lower cumulative energy intake than L12 animals from week 9 onward (Figure 1B), an effect that could be mainly explained by the decreased cumulative intake of CH, lipids, and protein from the ninth week (Figures 1C–E). The animals chronically exposed to the long photoperiod and fed the CAF for 7 weeks also showed significantly lower cumulative food intake than L12 rats at the end of the study (Figure 1B). Nevertheless, this effect was mainly attributed to the decreased cumulative CH intake observed from week 9 onward (Figure 1C), since no significant differences in lipid consumption were found (Figure 1D), and lower cumulative protein intake in L18 rats compared with L12 animals was only observed at the end of the study (Figure 1E). Moreover, both L6 and L18 rats displayed a significant decrease in cumulative fiber intake than L12 rats (Table 1). A detailed analysis of the consumption of the different food items included in the CAF diet revealed that L6 rats ate significantly less muffins and biscuits with cheese and pâté than L12 animals, whereas L18 rats consumed less chow than L12 animals and less carrots and more bacon than the L6 and L12 groups (Table 1). Both L6 and L18 animals consumed numerically lower amounts of milk with sugar—the food item that was consumed more by the rats—than L12 animals and, although the difference was not statistically significant, it contributed to the observed significant decrease in the cumulative CH intake (Table 1).

**Table 1:** Cumulative intake of different cafeteria diet food items and fiber at the end of the 7-week dietary study.

Cumulative intake	L6	L12	L18	
Chow (g)	31.5 ± 3.5	42.7 ± 5.6	29.5 ± 2.0	
Cheese and pâté biscuits (g)	50.4 ± 2.8	62.0 ± 4.4	55.4 ± 2.5	
Bacon (g)	21.7 ± 2.6^a^	23.7 ± 2.7^a^	34.1 ± 2.4^b^	*P*
Carrots (g)	48.3 ± 4.7^a^	53.3 ± 1.1^a^	35.0 ± 2.9^b^	*P*
Muffins (g)	38.2 ± 4.5^a^	49.3 ± 1.1^b^	50.7 ± 1.5^b^	*P*
Milk with sugar (mL)	399 ± 7	446 ± 34	371 ± 33	
Fiber (g)	4.33 ± 0.14^a^	5.31 ± 0.22^b^	4.14 ± 0.16^a^	*P*


*Male F344 rats were exposed to three different photoperiods for 11 weeks and fed a cafeteria diet for the last 7 weeks. Data are expressed as the mean ± SEM (*n* = 10). One-way ANOVA and Duncan’s *post hoc* tests were performed to compare differences between groups. Significant differences are represented by different letters (a, b). *P*, photoperiod effect.*

These changes in food intake-related parameters were not associated with an overall photoperiod effect on body weight gain (Figure 1A). Nevertheless, L18 animals displayed residually lower body weight gain compared with the L12 group at weeks 6, 7, 9, 10, and 11 (*p* < 0.05, Student’s *t*-test) and the same pattern was observed in L6 compared to L12 rats at weeks 6, 7, 8, and 9 (*p* < 0.05, Student’s *t*-test). In addition, at the end of the study, both L6 and L18 groups displayed lower body weight compared with L12 animals (*p* = 0.055 and *p* = 0.042, respectively, Student’s *t*-test) (Table 2).

**Table 2:** Biometric and serum parameters in F344 rats exposed to three different photoperiods for 11 weeks and fed a cafeteria diet for the last 7 weeks.

	L6	L12	L18	
**Biometric parameters**				
Initial body weight (g)	210 ± 4	221 ± 4	215 ± 4	
Final body weight (g)	407 ± 12	441 ± 11	411 ± 8	
Liver (g)	14.2 ± 0.5	14.8 ± 0.4	14.7 ± 0.4	
Skeletal muscle (g)	2.11 ± 0.05	2.23 ± 0.04	2.09 ± 0.03	
Testes (g)	3.00 ± 0.04	3.03 ± 0.07	2.92 ± 0.02	
Fat mass (g)	85.8 ± 3.3	84.4 ± 4.2	89.5 ± 3.8	
Fat mass (%)	21.5 ± 0.8	19.5 ± 0.9	22.0 ± 0.6	
Lean mass (g)	294 ± 8^a^	317 ± 6^b^	292 ± 5^a^	*P*
Lean mass (%)	73.7 ± 0.8^ab^	75.0 ± 0.8^b^	72.1 ± 0.5^a^	*P*
Lean/fat mass ratio	3.48 ± 0.18	3.92 ± 0.25	3.30 ± 0.12	
**Serum parameters**				
Glucose (mmol/L)	9.11 ± 0.27	9.73 ± 0.52	10.15 ± 0.26	
Insulin (ng/mL)	5.82 ± 0.24	6.52 ± 0.82	6.59 ± 0.47	
Glucagon (ng/mL)	2.53 ± 0.12	2.64 ± 0.06	2.63 ± 0.10	
Insulin:glucagon ratio	2.21 ± 0.13	2.53 ± 0.34	2.51 ± 0.21	
NEFAs (mmol/L)	1.52 ± 0.24	1.37 ± 0.13	1.57 ± 0.18	
Phospholipids (mmol/L)	3.87 ± 0.33	3.78 ± 0.22	4.18 ± 0.24	
Triglycerides (mmol/L)	5.30 ± 0.58	5.18 ± 0.39	5.28 ± 0.32	
Total cholesterol (mmol/L)	3.30 ± 0.41	3.33 ± 0.40	3.85 ± 0.41	


*Male F344 rats were exposed to three different photoperiods for 11 weeks and fed a cafeteria diet for the last 7 weeks. Data are expressed as the mean ± SEM (*n* = 10). One-way ANOVA and Duncan’s *post hoc* tests were performed to compare differences between groups. Significant differences are represented by different letters (a, b). *P*, photoperiod effect. The skeletal muscle weight represents the total weight of both the soleus and gastrocnemius muscles.*

### Hypothalamic mRNA Levels of Genes Related With Food Intake Control Were Vastly Regulated by Chronic Exposure to Both L6 and L18 Photoperiods

To shed light on the described photoperiod effects on caloric intake, we analyzed the mRNA levels of different genes related with the regulation of food intake in the hypothalamus of diet-induced obese rats. Intriguingly, we noted a sharp upregulation of the orexigenic neuropeptide Y (*Npy*) gene in both L6 and L18 groups compared with those exposed to the L12 photoperiod (81.2% and 78.3% higher, respectively), whereas no changes in the anorexigenic neuropeptides proopiomelanocortin (*Pomc*) and cocaine and amphetamine-regulated transcript (*Cart*) were observed (Figure 1F). In addition, the ghrelin receptor (*Ghsr*) mRNA levels were significantly increased in L6 and L18 animals compared to the L12 group (92% and 94.2% higher, respectively) (Figure 1F). No changes in the leptin receptor (*ObRb*) gene in response to different photoperiod exposures were noted (Figure 1F).

### CAF-Fed Obese Rats Exposed to Different Photoperiods Displayed Significant Differences in Body Composition

Compared to L12 animals, both L18 and L6 groups displayed lower absolute lean mass (Table 2). L18 animals also showed decreased skeletal muscle weight, significantly lower relative lean mass and, consequently, a lesser lean/fat mass ratio than their L12 counterparts (*p* < 0.05, Student’s *t*-test) (Table 2).

### Chronic Exposure to Different Photoperiods Modified Circulating Serum Glucose Levels and Some Serum Metabolites Analyzed by NMR

The analysis of serum showed that L18 photoperiod-exposed animals exhibited higher circulating glucose levels compared to the L6 group (*p* = 0.013, Student’s *t*-test), whereas no difference in either circulating insulin or glucagon levels was observed between groups (Table 2). In addition, only five circulating metabolites obtained by NMR analysis were significantly different between the photoperiod groups. Glutamate, glycine, and taurine were higher in L12 animals than the L6 and L18 groups (Supplementary Table 2). Moreover, L18 animals displayed higher circulating levels of proline compared to the L6 group and lower choline levels than their counterparts (Supplementary Table 2). This group also exhibited residually lower levels of 3-hydroxybutyrate (*p* = 0.05 versus L12 rats, Student’s *t*-test) and lactate (*p* = 0.052 and *p* = 0.048, compared to L6 and L12 animals, respectively, Student’s *t*-test) (Supplementary Table 2).

### Exposure to Different Day Lengths Significantly Modulated Whole-Body Substrate Oxidation, EE, and Locomotor Activity

**FIGURE 2:**
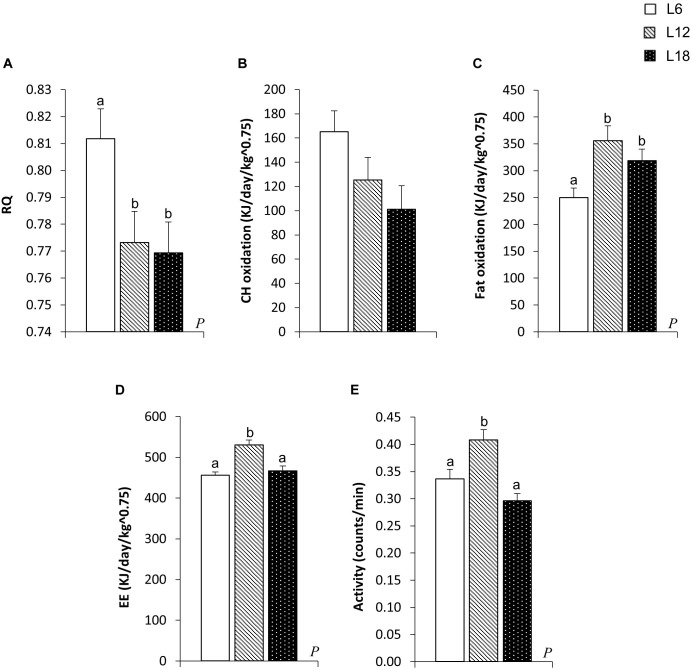
Respiratory quotient (RQ) **(A)**, carbohydrate (CH) oxidation **(B)**, fat oxidation **(C)**, energy expenditure (EE) **(D)**, and locomotor activity **(E)** in male F344 rats exposed to three different photoperiods for 11 weeks and fed a cafeteria diet for the last 7 weeks. Data are expressed as the mean ± SEM (*n* = 10). *P*, photoperiod effect. ^ab^Mean values with unlike letters were significantly different among groups (one-way ANOVA and Duncan’s *post hoc* test).

L6 animals displayed a significant increase in the RQ compared with their L12 and L18 counterparts (Figure 2A). Consequently, this group exhibited a residual increase in CH oxidation rates compared to L18 rats (*p* = 0.027, Student’s *t*-test) (Figure 2B) and a significant decrease in the fat oxidation levels compared with the L12 and L18 groups (29.8% and 21.5% lower, respectively) (Figure 2C). Thus, these findings revealed that exposure to a short photoperiod highly boosted the use of CH instead of lipids as an energy source. Both L6 and L18 animals showed lower EE and locomotor activity than L12 rats (Figures 2D,E).

### Skeletal Muscle Fatty Acid Uptake- and β-Oxidation-Related Genes Were Modulated by Exposure to Different Day Length Schedules

**FIGURE 3:**
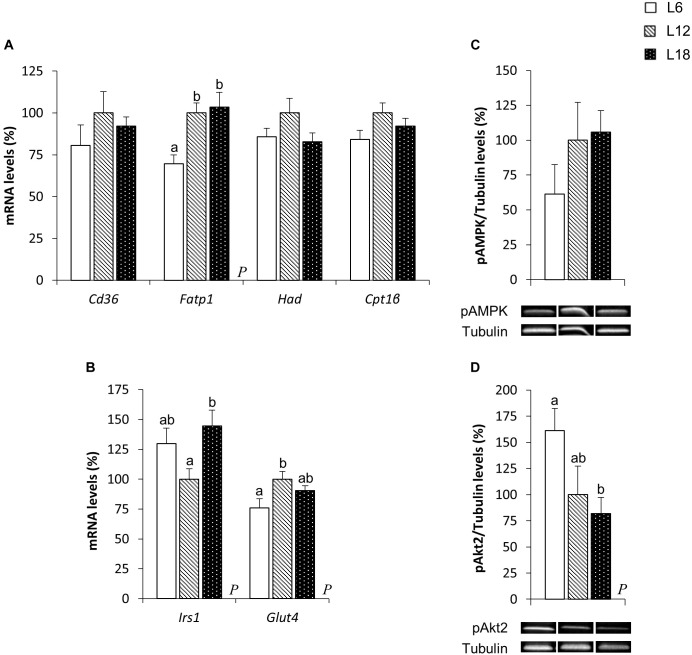
The mRNA expression of genes involved in lipid **(A)** and glucose **(B)** metabolism and pAMPK **(C)** and pAkt2 **(D)** protein levels in the soleus muscle of male F344 rats exposed to three different photoperiods for 11 weeks and fed a cafeteria diet for the last 7 weeks. Data are expressed as the mean ± SEM (*n* = 8). *P*, photoperiod effect. ^ab^Mean values with unlike letters were significantly different among groups (one-way ANOVA and Duncan’s *post hoc* test). *Cd36*, fatty acid translocase, homolog of CD36; *Cpt1*β, carnitine palmitoyltransferase 1 beta; *Fatp1*, fatty acid transport protein 1; *Glut4*, glucose transporter 4; *Had*, hydroxyacyl-CoA dehydrogenase; *Irs1*, insulin receptor substrate 1; pAkt2, phosphorylated Akt serine/threonine kinase 2; pAMPK, phosphorylated AMP-activated protein kinase.

**FIGURE 4:**
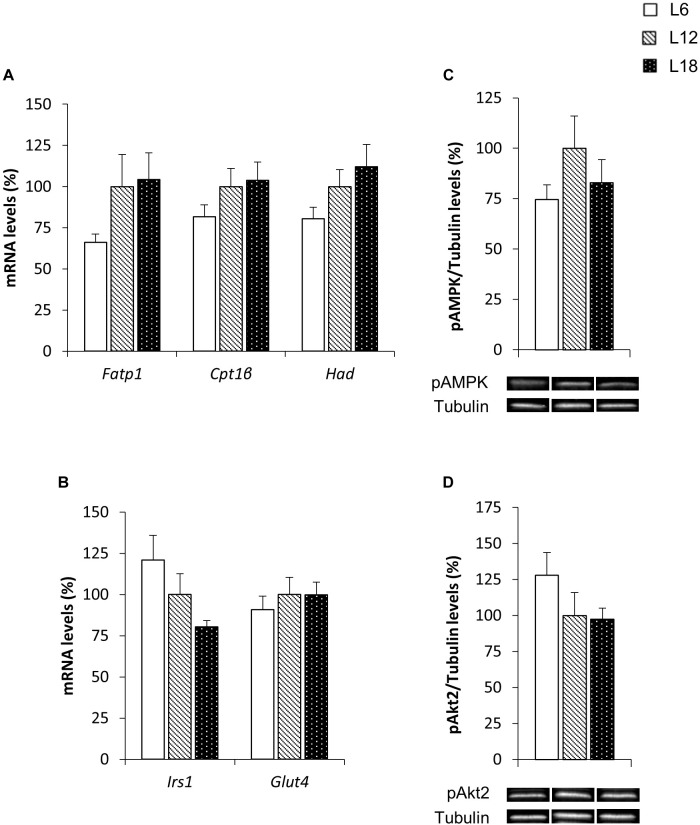
The mRNA expression of genes involved in lipid **(A)** and glucose **(B)** metabolism and pAMPK **(C)** and pAkt2 **(D)** protein levels in the gastrocnemius muscle of male F344 rats exposed to three different photoperiods for 11 weeks and fed a cafeteria diet for the last 7 weeks. Data are expressed as the mean ± SEM (*n* = 8). *P*, photoperiod effect. ^ab^Mean values with unlike letters were significantly different among groups (one-way ANOVA and Duncan’s *post hoc* test). The genes and proteins analyzed have already been described in Figure 3.

To elucidate the mechanisms involved in the photoperiodic modulation of whole-body substrate oxidation, we analyzed different lipid metabolism-related parameters in the skeletal muscle of CAF-fed obese rats. L6 photoperiod-exposed animals exhibited significant downregulation of the fatty acid transport protein 1 (*Fatp1*) mRNA levels compared to the L12 and L18 groups in the soleus muscle (30.3% and 32.6% lower, respectively) (Figure 3A). The expression of this gene showed very similar behavior in the gastrocnemius muscle (33.9% and 36.6% lower in L6 animals compared with L12 and L18 rats, respectively), although the differences were not statistically significant (*p* = 0.053 versus L18 rats, Student’s *t*-test) (Figure 4A). Moreover, L6 animals exhibited a clear trend toward decreased expression of the β-oxidation-related gene, hydroxyacyl-CoA dehydrogenase (*Had*), in the gastrocnemius muscle compared with the L18 group (*p* = 0.056, Student’s *t*-test) (Figure 4A) and numerically lower carnitine palmitoyltransferase 1 beta (*Cpt1*β) gene expression in the soleus muscle compared with L12 animals (*p* = 0.068, Student’s *t*-test) (Figure 3A). No differences in the phosphorylated levels of AMPK were found in either the soleus or gastrocnemius muscle among groups (Figures 3C, 4C).

### Exposure to Different Day Lengths Altered the Phosphorylated Levels of Akt2 and Other Glucose Metabolism-Related Genes in Both the Soleus and Gastrocnemius Muscles

To better understand the higher glycemia observed in L18 photoperiod-exposed animals, some parameters related with glucose homeostasis were analyzed in the skeletal muscle, which is considered the main contributor of postprandial glucose uptake in the organism (de Lange et al., 2007). L18 animals exhibited a sharp downregulation of the pAkt2 protein levels in the soleus muscle compared with the L6 group (49.2% lower) (Figure 3D), although no changes were observed in the pAkt2 levels in the gastrocnemius muscle (Figure 4D). Furthermore, residual downregulation of the *Irs1* mRNA levels was observed in the gastrocnemius muscle of L18 animals compared to L6 rats (*p* = 0.030, Student’s *t*-test) (Figure 4B). Intriguingly, in the soleus muscle, L18 animals displayed higher *Irs1* mRNA levels compared with L12 rats, and L6 animals exhibited a downregulation of the glucose transporter 4 (*Glut4*) gene (*p* < 0.05, Student’s *t*-test) (Figure 3B).

### Chronic Exposure to Different Photoperiods Induced Pronounced Changes in Key Genes Involved in Glucose and Lipid Homeostasis in the Liver

**FIGURE 5:**
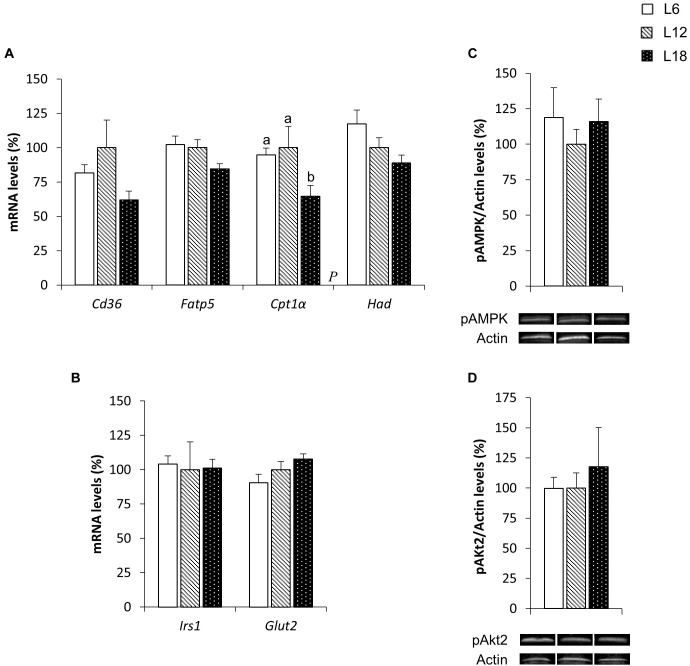
The mRNA expression of genes involved in lipid **(A)** and glucose **(B)** metabolism and pAMPK **(C)** and pAkt2 **(D)** protein levels in the liver of male F344 rats exposed to three different photoperiods for 11 weeks and fed a cafeteria diet for the last 7 weeks. Data are expressed as the mean ± SEM (*n* = 8). *P*, photoperiod effect. ^ab^Mean values with unlike letters were significantly different among groups (one-way ANOVA and Duncan’s *post hoc* test). *Cpt1*α, carnitine palmitoyltransferase 1 alpha; *Fatp5*, fatty acid transport protein 5; *Glut2*, glucose transporter 2. The rest of the genes and proteins analyzed have already been described in Figure 3.

In contrast to what was observed in the skeletal muscle, L18 photoperiod-exposed animals displayed a significant drop in the gene expression of several fatty acid uptake- and β-oxidation-related genes in the liver. Compared with the L6 and L12 groups, L18 animals exhibited residually lower fatty acid transport protein 5 (*Fatp5*) mRNA levels (*p* = 0.025 and *p* = 0.041, respectively, Student’s *t*-test) and a significant reduction in carnitine palmitoyltransferase 1 alpha (*Cpt1*α) gene expression (31.7% and 35.3% lower, respectively) (Figure 5A). This group also displayed lower fatty acid translocase, homolog of CD36 (*Cd36*) and *Had* mRNA levels than the L6 group (*p* = 0.045 and *p* = 0.027, respectively, Student’s *t*-test) (Figure 5A). The analysis of glucose metabolism-related genes revealed a residual increase in the glucose transporter 2 (*Glut2*) mRNA levels in L18 animals compared to those exposed to the L6 photoperiod (*p* = 0.032, Student’s *t*-test) (Figure 5B). No significant changes in either pAMPK or pAkt2 protein expression were observed among groups (Figures 5C,D).

### Hepatic and Muscular Core Clock Genes Were Significantly Modulated by Chronic Exposure to Different Photoperiods

**FIGURE 6:**
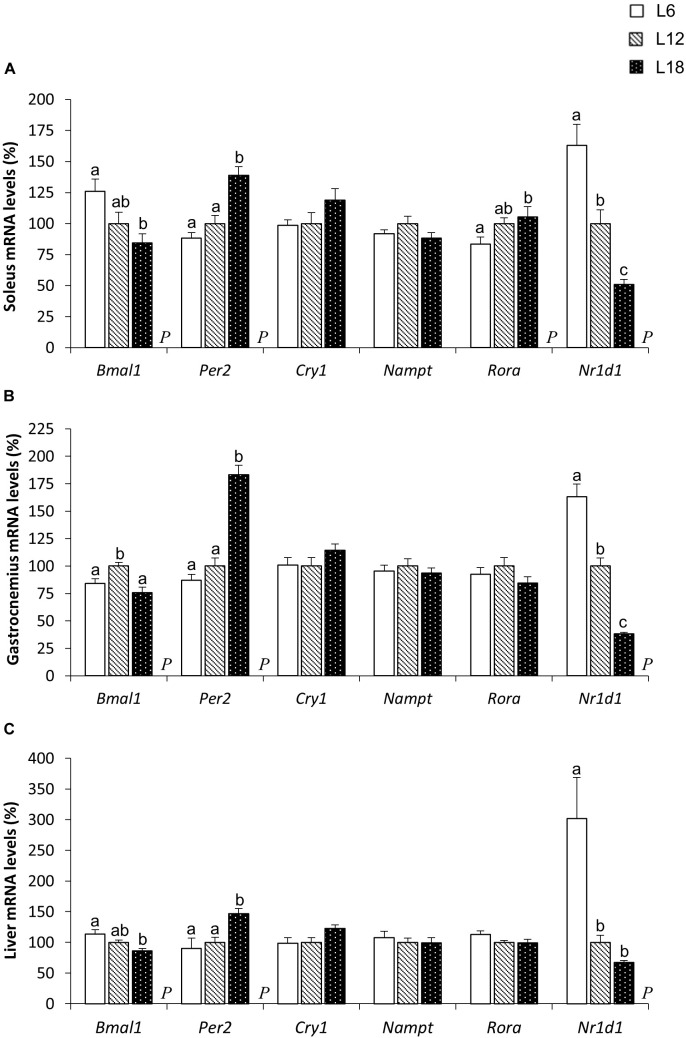
The mRNA expression of circadian core-clock genes in the soleus **(A)** and gastrocnemius **(B)** muscles and the liver **(C)** in male F344 rats exposed to three different photoperiods for 11 weeks and fed a cafeteria diet for the last 7 weeks. Data are expressed as the mean ± SEM (*n* = 8). *P*, photoperiod effect. ^abc^Mean values with unlike letters were significantly different among groups (one-way ANOVA and Duncan’s *post hoc* test). *Bmal1*, brain and muscle Arnt-like protein-1; *Cry1*, cryptochrome circadian clock 1; *Nampt*, nicotinamide phosphoribosyltransferase; *Nr1d1*, nuclear receptor subfamily 1, group D, member 1; *Per2*, period circadian clock 2; *Ror*α, RAR-related orphan receptor alpha.

A very similar expression pattern of genes related to circadian rhythms was observed in the liver and the soleus and gastrocnemius muscles among the three photoperiod-exposed groups. L18 animals displayed lower brain and muscle Arnt-like protein 1 (*Bmal1*) mRNA levels in the soleus muscle and the liver than the L6 group (Figures 6A,C) and exhibited an upregulation of the period circadian clock 2 (*Per2*) mRNA levels in both the soleus and gastrocnemius muscles and the liver compared to L6 and L12 photoperiod-exposed animals (Figures 6A–C). Similarly, cryptochrome circadian clock 1 (*Cry1*) gene expression levels were greater in L18 animals than in L6 and L12 rats, but these changes were only significant in the liver (*p* = 0.039 and *p* = 0.031, respectively, Student’s *t*-test) (Figure 6C). Finally, the gene that encodes the *Bmal1* transcription inhibitor nuclear receptor subfamily 1 group D member 1 (NR1D1) protein was sharply downregulated in the three analyzed tissues of L18 animals compared to the L12 group and to a greater extent compared with L6 animals (Figures 6A–C).

### The Multivariate Analysis Revealed a Marked Clustering Among the Different Photoperiod-Exposed CAF Groups

**FIGURE 7:**
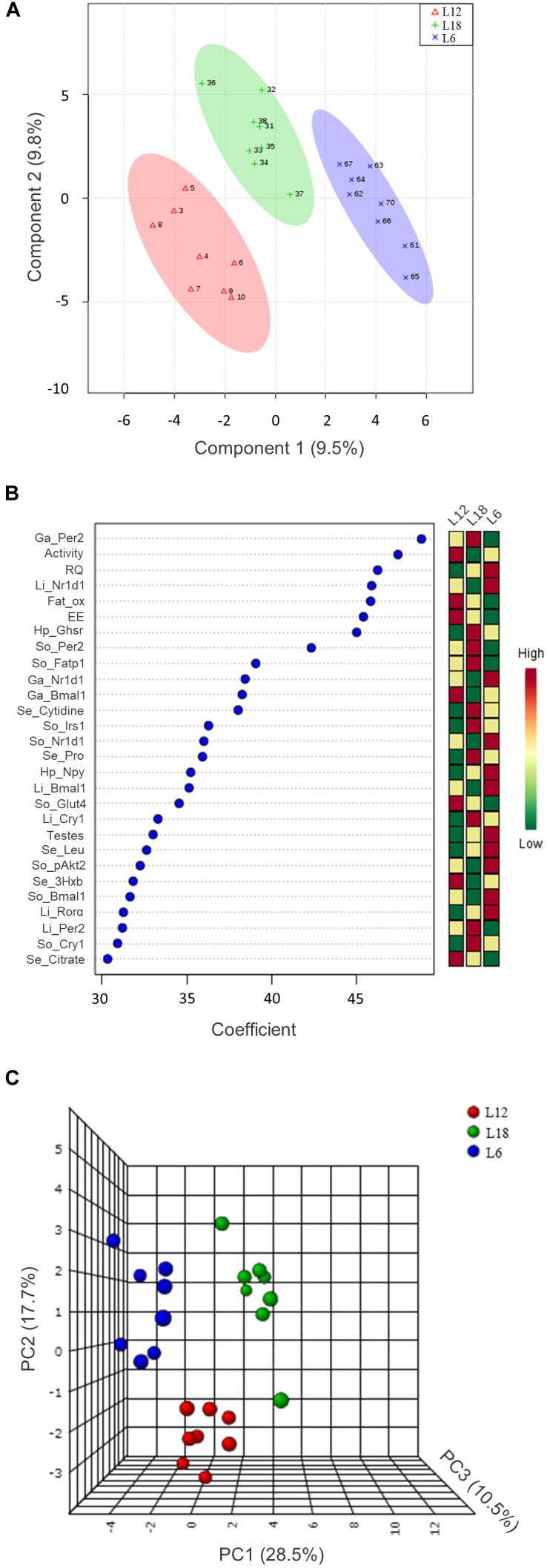
The 112 analyzed parameters were used to set up a PLS-DA predictive model **(A)**. The 28 variables with a coefficient mean higher than 30 (**(B)** were analyzed by PCA **(C)**. 3Hxb, 3-hydroxybutyrate; *Bmal1*, brain and muscle Arnt-like protein-1; *Cry1*, cryptochrome circadian clock 1; EE, energy expenditure; Fat_ox, fat oxidation; *Fatp1*, fatty acid transport protein 1; Ga, gastrocnemius muscle; *Ghsr*, ghrelin receptor; *Glut4*, glucose transporter 4; Hp, hypothalamus; *Irs1*, insulin receptor substrate 1; Leu, leucine; Li, liver; *Npy*, neuropeptide Y; *Nr1d1*, nuclear receptor subfamily 1, group D, member 1; pAkt2, phosphorylated Akt serine/threonine kinase 2; *Per2*, period circadian clock 2; Pro, proline; *Ror*α, RAR-related orphan receptor alpha; RQ, respiratory quotient; Se, serum; So, soleus muscle.

The 112 biometric, biochemical, physiological, and molecular parameters evaluated in the present study were used to set up a PLS-DA predictive model to detect marked clustering among the photoperiod groups (Figure 7A). After representing the three components’ scores, the quality parameters associated with this model were satisfactory. In this sense, the degree of fit of the model to the data, which is represented by *R*^2^, was 0.98. Furthermore, the cross-validation of this model (*Q*^2^) was 0.59; with a threshold of >0.4, this biological model is considered acceptable (Worley and Powers, 2013). Taking into account the good quality of this predictive model, we selected those variables with a coefficient mean higher than 30 to set up a PCA (Figure 7B).

After representing the 28 selected variables that exhibited a higher relevance in the separation of the three groups in the PCA, the 56.8% variance was explained. As shown in Figure 7C, clear clustering revealed a strong differential response of each group toward chronic exposure to different photoperiods. Among these variables, we mainly observed circadian rhythm-related genes (*Bmal1*, *Per2*, *Cry1*, *Ror*α, and *Nr1d1*) in the three analyzed tissues, hypothalamic orexigenic genes (*Npy* and *Ghsr*), glucose metabolism-related parameters (pAkt2 protein and *Glut4* and *Irs1* gene expression) in the skeletal muscle and physiologic parameters measured by indirect calorimetry analysis (RQ, EE, and fat oxidation).

## Discussion

In the present study, we demonstrated that chronic exposure to three different photoperiods (L6, L12, and L18) combined with the intake of the obesogenic diet CAF induced profound changes in a wide range of physiological and metabolic parameters in F344 rats. Briefly, we demonstrated that, in comparison with L12 animals, both L6 and L18 rats displayed lower final body weight and cumulative caloric intake, increased hypothalamic mRNA levels of the orexigenic genes *Npy* and *Ghsr*, and decreased EE and locomotor activity. Nevertheless, despite these common effects observed in both L6 and L18 CAF-fed animals, most of the physiological and metabolic changes reported in the present study were found between these two groups. Thus, we observed: (1) a higher preference for fat-rich food items in L18 rats; (2) higher circulating glucose levels in L18 animals, which were accompanied by a downregulation of the phosphorylated levels of Akt2 in the soleus muscle and lower *Irs1* mRNA levels in the gastrocnemius muscle; (3) reduced whole-body lipid utilization in L6 animals, which was supported by the downregulation of fatty acid transporters and β-oxidation-related genes in both skeletal muscles; and (4) profound changes in the hepatic and muscular expression of the circadian rhythm-related genes *Cry1*, *Bmal1*, *Per2*, and *Nr1d1.* In addition, the multivariate analysis carried out with 112 parameters, including biometric- and food intake-related parameters as well as biochemical and molecular variables in the blood, liver, skeletal muscles, and hypothalamus, revealed a clear clustering among the three photoperiod groups, reinforcing the relevance of changes in seasonal day length in physiology and metabolism in the obese state.

Numerous studies have already shown a marked photoperiod effect on several physiologic, behavioral, and reproductive parameters in healthy F344 rats exposed to different day length schedules (Heideman and Sylvester, 1997; Heideman et al., 2000; Shoemaker and Heideman, 2002; Ross et al., 2011; Tavolaro et al., 2015). In this sense, our group previously reported that, under normoweight conditions, chronic exposure to different photoperiods induced relevant changes in a variety of glucose and lipid metabolism-related parameters in this model (Mariné-Casadó et al., 2018). However, in terms of biometric and reproductive parameters, normoweight F344 rats held under an SD photoperiod exhibited a significant photorefractory response after 14 weeks, not displaying the widely described short photoperiod-like regressive phenotype in body weight, body composition, and testes size (Shoemaker and Heideman, 2002; Tavolaro et al., 2015) compared to animals exposed to an LD photoperiod (Mariné-Casadó et al., 2018). In the present study, an adaptation period of 4 weeks to the different photoperiods did not manifest into changes in body weight or food intake in F344 rats fed an STD, which was in agreement with our previous findings (Mariné-Casadó et al., 2018) but clearly differed from other studies in which marked variations in cumulative food intake (15 days) and body weight gain (20 days) were rapidly reported after exposure to different day lengths (Tavolaro et al., 2015). However, the inclusion of the CAF for the subsequent 7 weeks provoked slight but significant decreases of body weight gain and final body weight in both L6 and L18 animals, which could be mostly attributed to the decreased cumulative energy intake displayed by both groups of rats over the course of the CAF intervention. Intriguingly, this lower caloric intake was accompanied by a sharp upregulation of the hypothalamic expression of the gene encoding the orexigenic neuropeptide NPY, which is one of the main enhancers of appetite (Sasaki, 2017), and by increased mRNA levels of the receptor of ghrelin, a gastric orexigenic hormone that acts through the activation of NPY neurons (Nakazato et al., 2001). One possible explanation for these contradictory results could be a differential feeding state among groups at sacrifice, which could markedly regulate leptin and ghrelin systems (Sánchez et al., 2010). However, the lack of variation in the circulating insulin and glucagon levels among groups strongly suggests that all animals were sacrificed under a very similar feeding status and, therefore, it seems very unlikely that differences in this parameter could account for the observed findings. Body weight is mainly determined by the balance between EE and energy intake (Sasaki, 2017). Thus, at first glance, the lower energy intake observed in both L6 and L18 rats could be understood as an adaptive response to fit with the decreased EE observed in these groups in an attempt to maintain an optimal weight. Nevertheless, these decreases in both caloric intake and EE were translated into a progressive loss of weight gain, suggesting an impairment of the mechanisms involved in body weight control in animals held under the SD and LD photoperiods. In this context, it is plausible to hypothesize that the activation of the orexigenic pathways could be a compensatory mechanism used to promote food intake in an attempt to counteract the lower body weight increase and, therefore, to recover the energy balance in L6 and L18 rats. One limitation of the obtained data is the fact that they only represent the endpoint of the study. Additional studies in which the animals are sacrificed at a later time point would be of great value to shed more light on this issue.

In addition to changes in energy intake, photoperiod clearly altered food preferences and macronutrient intake. Thus, the higher preference for some fat-rich solid food items included in the CAF (bacon and muffins) observed in L18 animals compared with L6 rats may explain the higher lipid consumption of animals exposed to a long-duration day than those held under the SD photoperiod, despite the fact that both groups attained a very similar overall caloric intake. Furthermore, the increased consumption of bacon reported in L18 rats compared with L12 animals and the similar muffin intake reported in both groups over the course of the study may account for the lack of significant differences in cumulative lipid intake between ND and LD photoperiod-exposed animals. The increased fat intake and higher preference for fat-rich foodstuffs observed in L18 rats compared with L6 animals is not consistent with the results obtained by Togo et al. (2012), which reported that F344 rats exposed to a long photoperiod (16 h of light/day) during 3 weeks displayed a significant preference for a low-fat, high-CH diet than for a high-fat, low-CH diet, although no differences in protein and fat intake were reported compared to animals exposed to a short photoperiod (8 h of light/day) (Togo et al., 2012). One possible explanation for these discrepancies could rely on the different experimental designs in terms of study length and/or diet compositions. Further analyses related with the hedonic regulation of food intake, such as hippocampal expression of genes and proteins involved in dopaminergic pathways [dopamine receptor D5 (DRD5) and dopamine transporter (DAT)] (Sanchez-Hernandez et al., 2015) and more specific tests concerning feeding behavior, such as the two-bottle preference test (Sánchez et al., 2008), would be of high relevance to gain further insight into the relevance of photoperiod exposure on lipid and CH preferences in CAF-fed rats.

The lower cumulative protein intake observed in both L6 and L18 animals could explain, at least in part, the decrease in absolute lean mass observed in both groups (Tirapegui et al., 2012). Since quantitative magnetic resonance analysis of lean mass provides a precise measurement of muscle mass (Taicher et al., 2003) and L18 animals showed lower skeletal muscle weight than L12 rats, it seems clear that chronic exposure to the LD photoperiod slowed down muscle mass accretion. Although skeletal muscle accounts for ≃80% of the postprandial circulating glucose uptake (de Lange et al., 2007), this finding was not translated into significant changes in the circulating levels of glucose between animals held under ND and LD photoperiods. Nevertheless, we reported a significant increase in blood glucose of L18 animals compared with L6 rats, which could be partly explained by the significant downregulation of the phosphorylated levels of the downstream postreceptor target of insulin Akt2 (Gonzalez and McGraw, 2009) and lower expression of the insulin signaling-related gene *Irs1*—involved in the activation of Akt2 (Long et al., 2011)—observed in the soleus and gastrocnemius muscle, respectively, of the group exposed to the long-duration day. Although we recently reported that chronic exposure to 18 h of light induced an increase in circulating glucose levels compared with L12 photoperiod-exposed normoweight F344 rats, these results are not in agreement with the idea that short-duration days are more associated with impairments in glucose metabolism and insulin signaling than long photoperiods, as was previously described by our group in F344 rats (Mariné-Casadó et al., 2018) and by Tashiro et al. (2017) in C57BL/6J mice. However, in the present study, the lack of significant changes in *Irs1* and *Glut4* gene expression in the soleus muscle between L18 and L6 animals and the increased mRNA levels of the hepatic glucose transporter *Glut2* in the L18 group do not fully support the hypothesis of a lower insulin sensitivity phenotype in LD-exposed CAF-fed animals. Taking into account that gene expression data do not always match the protein levels, further analyses concerning GLUT4 translocation and the levels of other proteins involved in glucose and insulin signaling pathways are needed. Nevertheless, to the best of our knowledge, this is the first study to report a clear interaction between an obesogenic diet and day length seasonal variations in the regulation of glucose homeostasis, although the mechanisms involved in these diet-dependent photoperiod effects deserve further research.

We previously reported that normoweight L6 photoperiod-exposed animals displayed increased circulating NEFAs levels, which were accompanied by a downregulation of the mRNA levels of the gene encoding the fatty acid transporter CD36 in the soleus muscle and liver and by decreased expression of the β-oxidation-related genes, *Cpt1*β and *Had*, in the soleus muscle (Mariné-Casadó et al., 2018). In the present study, exposure to the L6 photoperiod combined with CAF intervention also produced profound changes in lipid metabolism, as illustrated by the reduced whole-body fat oxidation rates in L6 animals compared with L12 and L18 rats. The lower lipid substrate utilization observed in this group could be mainly attributed to its significantly lower lipid intake, decreased mRNA levels of the fatty acid transporter gene, *Fatp1*, observed in both the soleus and gastrocnemius muscles, and the clear trend toward lower mRNA levels of the β-oxidation-related genes, *Cpt1*β and *Had*, observed in the soleus and gastrocnemius muscle, respectively. However, in contrast to what was observed in normoweight rats, the molecular changes related with fatty acid metabolism observed in the skeletal muscles of L6 CAF-fed animals were not translated into elevated circulating NEFAs. One possible explanation for these results could be the enhancement of fatty acid transport and β-oxidation pathways observed in the liver, which is illustrated by the upregulation of *Cd36*, *Fatp5*, *Cpt1*α, and *Had* mRNA levels observed in L6 animals compared to L18 rats. This liver-specific response could be explained as a compensatory action addressed to increase the hepatic fatty acid supply to maintain fatty acid homeostasis and, therefore, to avoid the rise of blood NEFAs. Nevertheless, further analyses of genes and proteins involved in fatty acid metabolism in the liver and peripheral tissues that significantly contribute to fatty acid uptake and β-oxidation, such as white adipose tissue (Luo and Liu, 2016), would be useful to corroborate this hypothesis.

We previously described that chronic exposure to different photoperiods in normoweight rats modulated the circadian core-clock transcriptional machinery, inducing profound changes in the gene expression levels of the major circadian clock transcriptional activator *Bmal1* (Robinson and Reddy, 2014), its product, *Per2*, and its inhibitor, *Nr1d1*, in the liver and both the soleus and gastrocnemius muscles in L18 animals (Mariné-Casadó et al., 2018). Taking into account that several studies have proven that these genes are markedly implicated in the regulation of feeding behavior (Sasaki, 2017) and different metabolic pathways, such as gluconeogenesis or fatty acid β-oxidation (Ribas-Latre and Eckel-Mahan, 2016), we speculate that the circadian rhythm-related gene expression changes observed in normoweight L18 rats could account, at least in part, to the physiologic and metabolic changes observed in this group, despite the limitation of only performing a single-point measurement of each gene (ZT1-2) (Mariné-Casadó et al., 2018). In the present study, CAF-fed rats chronically exposed to different day lengths displayed a very similar gene expression pattern of core-clock genes than those reported in our previous study (Mariné-Casadó et al., 2018), suggesting that the CAF did not alter the circadian transcriptional machinery regulation. Remarkably, despite the fact that the hepatic and muscular gene expression profiles of circadian rhythm-related genes were very similar, we observed a very different response in genes related to fatty acid uptake and oxidation between the liver and skeletal muscles. Thus, although the marked variations in the mRNA levels of *Nr1d1* and *Per2* in the liver and skeletal muscles may partly explain the differences in physiologic and metabolic parameters observed among the different photoperiod groups, the abovementioned results would make it difficult to establish the relevance of these gene expression changes on the observed effects. Therefore, further analyses performed at different time points throughout a 24-h period are needed to shed more light on this issue.

## Conclusion

We demonstrated that the consumption of a CAF triggered marked variations in outputs related with body weight regulation, feeding behavior, and metabolism in F344 rats chronically exposed to different photoperiods. Relevantly, we observed a very similar behavior concerning caloric intake and biometric parameters in obese rats exposed to both short and long photoperiods, describing a decrease in body weight gain, lean mass, energy intake, and EE compared with animals exposed to 12 h of light. These changes were accompanied by a significant increase in the hypothalamic expression of the orexigenic genes *Npy* and *Ghsr*, in both groups of animals, which could be interpreted as a mechanism for increasing food intake to restore body weight homeostasis. Nevertheless, these common outputs between L6 and L18 animals were associated with different metabolic adaptations, as was illustrated by (1) the higher circulating glucose levels observed in animals held under the long photoperiod than L6 rats, which would be partly attributed to decreased pAkt2 protein levels and *Irs1* mRNA levels in the soleus and gastrocnemius muscles, respectively; and (2) the lower whole-body fat utilization in L6 animals, an effect that could be partly associated with decreased fat intake and the downregulation of fatty acid uptake- and β-oxidation-related genes in skeletal muscles. Since a seasonal cycle of food consumption in humans has been described (Kräuchi and Wirz-Justice, 1988; Pijl et al., 2001), our study could contribute to highlight the relevance of the intake of highly palatable and energy dense foods prevalent in Western societies in the physiological and metabolic adaptations that occur in response to seasonal variations of day length, especially in diseases associated with changes in food intake frequency and preference, such as obesity (Sasaki, 2017) and SAD (Kräuchi and Wirz-Justice, 1988). The impact of these findings on human physiology and health deserves further research.

## Ethics Statement

This study was carried out in accordance with the recommendations of the guidelines for the use and care of laboratory animals of the Universitat Rovira i Virgili. The protocol was approved by the Bioethics Committee of this university.

## Author Contributions

CB, LA, AC, and JdB designed the studies. RM-C, CD-C, AC, and JdB performed the experiments and analyzed the data. RM-C, AC, and LA wrote the manuscript. All the authors read, discussed, and approved the final version of the manuscript.

## Conflict of Interest Statement

The authors declare that the research was conducted in the absence of any commercial or financial relationships that could be construed as a potential conflict of interest.
