# Supplementary material for: Intake of an Obesogenic Cafeteria Diet Affects Body Weight, Feeding Behavior, and Glucose and Lipid Metabolism in a Photoperiod-Dependent Manner in F344 Rats
Source: Front Physiol. 2018 Nov 26;9:1639. doi: 10.3389/fphys.2018.01639 (PMC6275206; doi:10.3389/fphys.2018.01639)
Supplement: Supplementary file 1 [file Table_1.DOC]

Supplementary Material

Intake of an obesogenic cafeteria diet affects body weight, feeding behavior and glucose and lipid metabolism in a photoperiod-dependent manner in F344 rats

**Roger Mariné-Casadó^1^, Cristina Domenech-Coca^2^, Josep Maria del Bas^1^, Cinta Bladé^2^, Lluís Arola^1,2*^, Antoni Caimari^1^**

*** Correspondence:** Prof. Lluís Arola: [lluis.arola@eurecat.org](mailto:lluis.arola@eurecat.org)

**Supplementary Table 1. Nucleotide sequences of primers used for real time quantitative PCR.**

| **Gene** | **Forward primer**  **(5’ to 3’)** | | **Reverse primer**  **(5’ to 3’)** | **Tissue** |
| --- | --- | --- | --- | --- |
| *β-actin* | TACAGCTTCACCACCACAGC | | TCTCCAGGGAGGAAGAGGAT | *L* |
| *Bmal1* | GTAGATCAGAGGGCGACGGCTA | | CTTGTCTGTAAAACTTGCCTGTGAC | *G, L, S* |
| *Cart* | AGAAGAAGTACGGCCAAGTCC | | CACACAGCTTCCCGATCC | *H* |
| *Cd36* | | GTCCTGGCTGTGTTTGGA | GCTCAAAGATGGCTCCATTG | *L, S* |
| *Cpt1α* | | GCTCGCACATTACAAGGACAT | TGGACACCACATAGAGGCAG | *L* |
| *Cpt1β* | | GCAAACTGGACCGAGAAGAG | CCTTGAAGAAGCGACCTTTG | *G, S* |
| *Cry1* | | TGGAAGGTATGCGTGTCCTC | TCCAGGAGAACCTCCTCACG | *G, L, S* |
| *Fatp1* | | TGCTCAAGTTCTGCTCTGGA | CATGCTGTAGGAATGGTGGC | *G, S* |
| *Fatp5* | | CCTGCCAAGCTTCGTGCTAAT | GCTCATGTGATAGGATGGCTGG | *L* |
| *Ghsr* | | TCAGCCAGTACTGCAACCTG | GGAGAGATGGGATGTGCTGT | *H* |
| *Glut2* | | AGTCACACCAGCACATACGA | TGGCTTTGATCCTTCCGAGT | *L* |
| *Glut4* | | CCATTGCTTCTGGCTATCAC | TCCGTTTCTCATCCTTCAGC | *G, S* |
| *Had* | | ATCGTGAACCGTCTCTTGGT | AGGACTGGGCTGAAATAAGG | *G, L, S* |
| *Hprt* | | TCCCAGCGTCGTGATTAGTGA | CCTTCATGACATCTCGAGCAAG | *G, L, S* |
| *Irs1* | | CTACACCCGAGACGAACACT | TAACCTGCCAGACCTCCTTG | *G, L, S* |
| *Nampt* | | CTCTTCACAAGAGACTGCCG | TTCATGGTCTTTCCCCCACG | *G, L, S* |
| *Npy* | | TGGACTGACCCTCGCTCTAT | GTGTCTCAGGGCTGGATCTC | *H* |
| *Nr1d1* | | ACAGCTGACACCACCCAGATC | CATGGGCATAGGTGAAGATTTCT | *G, L, S* |
| *ObRb* | | AGCCAAACAAAAGCACCATT | TCCTGAGCCATCCAGTCTCT | *H* |
| *Per2* | | CGGACCTGGCTTCAGTTCAT | AGGATCCAAGAACGGCACAG | *G, L, S* |
| *Pomc* | | CCTGTGAAGGTGTACCCCAATGTC | CACGTTCTTGATGATGGCGTTC | *H* |
| *Ppia* | | CCAAACACAAATGGTTCCCAGT | ATTCCTGGACCCAAAACGCT | *G, S* |
| *Rorα* | | CCCGATGTCTTCAAATCCTTAGG | TCAGTCAGATGCATAGAACACAAACTC | *G, L, S* |
| *Tfrc* | | ATCATCAAGCAGCTGAGCCAG | CTCGCCAGACTTTGCTGAATTT | *S* |

The table shows the nucleotide sequences of primers used for PCR amplification. Primer pairs for PCR were designed using Primer3 software and the sequence information were obtained from Genbank. *β-actin*, actin beta; *Bmal1,* brain and muscle Arnt-like protein-1; *Cart,* cocaine and amphetamine-regulated transcript; *Cd36*, fatty acid translocase, homologue of CD36; *Cpt1α*, carnitine palmitoyltransferase 1 alpha; *Cpt1β*, carnitine palmitoyltransferase 1 beta; *Cry1,* cryptochrome circadian clock 1; *Fatp1*, fatty acid transport protein 1; *Fatp5*, fatty acid transport protein 5; *Ghsr*, ghrelin receptor; *Glut2*, glucose transporter 2; *Glut4,* glucose transporter 4; *Had*, hydroxyacyl-CoA dehydrogenase; *Hprt*, hypoxanthine guanine phosphoribosyl transferase; *Irs1,* insulin receptor substrate 1; *Nampt,* nicotinamide phosphoribosyltransferase*; Npy,* neuropeptide Y; *Nr1d1,* nuclear receptor subfamily 1, group D, member 1*; ObRb,* long-form leptin receptor; *Per2,* period circadian clock 2*; Pomc*, proopiomelanocortin; *Ppia*, peptidylprolyl isomerase A; *Rorα*, RAR-related orphan receptor A; *Tfrc*, transferrin receptor. Gene expression levels were analyzed in the hypothalamus (H), liver (L) and the gastrocnemius (G) and soleus (S) muscles.
