# Supplementary material for: Intake of an Obesogenic Cafeteria Diet Affects Body Weight, Feeding Behavior, and Glucose and Lipid Metabolism in a Photoperiod-Dependent Manner in F344 Rats
Source: Front Physiol. 2018 Nov 26;9:1639. doi: 10.3389/fphys.2018.01639 (PMC6275206; doi:10.3389/fphys.2018.01639)
Supplement: Supplementary file 2 [file Table_2.DOC]

Supplementary Material

Intake of an obesogenic cafeteria diet affects body weight, feeding behavior and glucose and lipid metabolism in a photoperiod-dependent manner in F344 rats

**Roger Mariné-Casadó^1^, Cristina Domenech-Coca^2^, Josep Maria del Bas^1^, Cinta Bladé^2^, Lluís Arola^1,2*^, Antoni Caimari^1^**

*** Correspondence:** Prof. Lluís Arola: [lluis.arola@eurecat.org](mailto:lluis.arola@eurecat.org)

**Supplementary Table 2. Concentration of representative serum metabolites analyzed by Nuclear Magnetic Resonance in F344 rats exposed to three different photoperiods for 11 weeks and fed a cafeteria diet for the last 7 weeks.**

| **Metabolite concentration (μmol/L)** | **L6** | **L12** | **L18** |  |
| --- | --- | --- | --- | --- |
| 3-Hydroxybutyrate | 61.7 ± 5.8 | 65.4 ± 4.7 | 53.2 ± 3.2 |  |
| Acetate | 64.3 ± 2.5 | 67.8 ± 3.1 | 67.6 ± 3.2 |  |
| Alanine | 166.6 ± 8.8 | 170.5 ± 5.6 | 177.5 ± 7.8 |  |
| Creatine | 103.2 ± 5.7 | 116.6 ± 4.4 | 109.5 ± 6.7 |  |
| Choline | 6.3 ± 0.3 ^a^ | 5.9 ± 0.1 ^a^ | 5.4 ± 0.1 ^b^ | *P* |
| Formate | 23.9 ± 1.1 | 25.0 ± 0.5 | 25.1 ± 1.3 |  |
| Glutamine | 171.4 ± 4.6 | 177.2 ± 4.5 | 176.1 ± 5.7 |  |
| Glutamate | 39.2 ± 1.3 ^a^ | 44.7 ± 2.2 ^b^ | 39.4 ± 1.5 ^a^ | *P* |
| Glycerophosphocholine | 34.9 ± 5.2 | 37.0 ± 5.5 | 27.8 ± 2.9 |  |
| Glycine | 64.4 ± 2.7 ^a^ | 71.3 ± 1.1 ^b^ | 65.3 ± 1.8 ^a^ | *P* |
| Histidine | 22.5 ± 0.7 | 23.4 ± 0.8 | 22.2 ± 0.9 |  |
| Isoleucine | 34.8 ± 1.9 | 32.3 ± 0.9 | 36.5 ± 3.1 |  |
| Lactate | 1259 ± 77 | 1314 ± 107 | 1048 ± 63 |  |
| Leucine | 31.6 ± 1.8 | 27.9 ± 0.6 | 32.7 ± 2.9 |  |
| Lysine | 124.8 ± 4.8 | 119.6 ± 3.9 | 127.1 ± 8.7 |  |
| Phenylalanine | 29.2 ± 0.7 | 31.1 ± 0.8 | 29.0 ± 0.7 |  |
| Proline | 52.6 ± 3.3 ^a^ | 55.3 ± 2.5 ^ab^ | 62.2 ± 1.9 ^b^ | *P* |
| Pyruvate | 22.9 ± 0.9 | 26.4 ± 2.2 | 23.2 ± 1.8 |  |
| Serine | 85.5 ± 2.2 | 85.5 ± 3.0 | 85.4 ± 3.1 |  |
| Taurine | 353 ± 10 ^a^ | 391 ± 17 ^b^ | 340 ± 10 ^a^ | *P* |
| Threonine | 52.8 ± 2.9 | 52.3 ± 2.7 | 52.6 ± 3.9 |  |
| Tryptophan | 44.1 ± 1.5 | 45.0 ± 1.3 | 43.5 ± 1.8 |  |
| Tyrosine | 33.1 ± 0.8 | 34.1 ± 1.3 | 30.6 ± 1.2 |  |
| Valine | 49.0 ± 2.2 | 46.7 ± 1.9 | 51.1 ± 4.3 |  |

Male Fischer 344 rats were exposed to three different photoperiods for 11 weeks and fed a cafeteria diet for the last 7 weeks. Data are expressed as the mean ± SEM (n=10). All the metabolites were obtained by performing a nuclear magnetic resonance (NMR) analysis. One-way ANOVA and Duncan’s post hoc tests were performed to compare the values between groups and significant differences were represented with different letters (a, b). *P*, photoperiod effect.
